# Association of social disengagement with health status and all-cause mortality among community-dwelling older adults: evidence from the Otassha study

**DOI:** 10.1038/s41598-022-22609-y

**Published:** 2022-10-26

**Authors:** Manami Ejiri, Hisashi Kawai, Kumiko Ito, Hirohiko Hirano, Yoshinori Fujiwara, Kazushige Ihara, Hunkyung Kim, Shuichi Obuchi

**Affiliations:** 1 Tokyo Metropolitan Institute of Gerontology, 35-2 Sakae-Cho, Itabashi-Ku, Tokyo 173-0015 Japan; 2 Department of Social Medicine, Hirosaki University School of Medicine, 5 Zaifu-Cho, Hirosaki City, Aomori 036-8560 Japan

**Keywords:** Geriatrics, Public health

## Abstract

This study examined the impact of disengagement on health status and mortality among community-dwelling older adults in Japan. Disengagement from society was operationally defined as dropping out of a longitudinal survey. A follow-up mail survey was conducted, in 2014, among respondents (n = 3696) of the baseline mail survey. Step-by-step follow-up surveys (FLs), including simplified mail, postcard, and home-visit surveys, were sent to participants who did not respond. Disengagement levels were defined according to the response to the FLs as zero (mail survey), low (simplified mail survey), middle (postcard survey), high (home-visit survey), and highest (non-responders to the home-visit survey). After adjusting for health status at baseline, the proportion of respondents self-rated as “not healthy” during FLs was significantly higher in the high-level than in the zero-level group. The proportion of respondents reporting a “once a week or less” frequency of going outdoors during FLs was significantly higher in the low-, middle-, and high-level groups than in the zero-level group. Mortality rates were significantly higher in the high and highest levels than in the zero-level group. Higher disengagement levels increased the risk of lower health status and mortality, suggesting an urgent need to prevent societal disengagement among older adults.

## Introduction

In disengagement theory, advocated by Cumming and Henry^1^, successful aging means “the acceptance and the desire for a process of disengagement from active life”^2^. According to this theory, aging is considered to be a process of withdrawal and disengagement, in which people’s relationships with others that make up their society are inexorably reduced. The adaptive behavior of disengagement in response to the loss of roles, such as occupation or parenthood, allows older adults to maintain a sense of self-worth, while disengagement among older adults is considered to have positive consequences for both society and older adults. However, life expectancy has recently increased, and the social system has changed. Many studies have revealed that active social participation in older age contributes to healthy aging, such as independence of Instrumental Activities of Daily Living (IADL)^3^, prevention of social isolation and cognitive decline^4,5^, and decline in mortality^6^. Thus, maintaining participation in society, rather than disengagement, is important for achieving successful aging.

Cooperation in research is a type of social participation/contribution. Therefore, withdrawal from a survey can be operationally defined as disengagement from society. Numerous studies have reported the characteristics of dropouts in longitudinal surveys of older adults. The participants who dropped out from surveys were relatively older^7–16^, had lower socioeconomic status^7,11,14^, had IADL/activities of daily living (ADL) disability^11,13,16^, showed lower self-rated health^7,9,14–16^, exhibited cognitive decline^8–10,16,17^ and depressive symptoms^9,14,17^, had poor social networks^8,9,15,16^, and showed lower gait functions^9,17,18^ at baseline surveys (BLs) compared to those who did not drop out. These findings suggest that older adults who disengaged from society had lower physical, mental, and social health statuses than those who did not. Thus, to prevent societal disengagement, it is important to maintain these health statuses.

Previously, Kawai et al. re-approached the dropouts and conducted a step-by-step follow-up survey (FL) to identify participants’ levels of disengagement from society^19^. For those who did not respond to the mail FL, simplified and easier-to-answer surveys, such as a simplified mail survey, postcard survey, and home-visit surveys, were conducted in sequence. The level of disengagement from society was defined based on the FLs at which responses were obtained^19^. They found that some older adults gradually disengaged from society and that characteristics, such as IADL, social participation, self-rated health, socioeconomic status, and social isolation at BL, differed by the disengagement level^19^. The characteristics of older adults who dropped out of the surveys were similar to the predictors of decreased participation in older men reported by Fairhall et al., such as higher age, chronic diseases, cognitive decline, low ADL, and frailty^9^, which reflect the characteristics of those with reduced participation and disengagement from society. However, the relationship between the level of disengagement and subsequent health status and its impact on mortality risk has not yet been clarified.

From the perspective of gerontology, it is beneficial to reveal the health status and mortality risk of those who are gradually disengaging from society to strengthen the knowledge of successful aging. Additionally, from the public health perspective, the results will provide evidence that it is beneficial for the follow-up of older adults who have ceased using health and welfare services or health checkups. This study aimed to clarify the relationship between the societal disengagement levels and health status, including death.

## Results

### Characteristics of study participants

The characteristics of the participants at BL and FLs stratified by disengagement level are shown in Tables 1 and 2. The proportion of those who were unhealthy and those who went outdoors once a week or less increased as the disengagement level increased. The proportions of respondents who answered not healthy at FLs at each disengagement level were 17.8%, 21.9%, 29.5%, and 33.3% at the zero, low, middle, and high levels, respectively. The proportion of respondents who went outdoors once a week or less at FLs were 6.8%, 11.3%, 15.8%, and 16.7% at the zero, low, middle, and high levels, respectively. The number of deaths among participants of each level was 212 (10.0%) at the zero level, 43 (10.8%) at the low level, 31 (14.9%) at the middle level, 21 (29.2%) at the high level, and 17 (21.3%) at the highest level.

**Table 1:** Characteristics of participants at baseline survey by disengagement levels (N = 3262).

	Zero-level (n = 2381)	Low-level (n = 462)	Middle-level (n = 234)	High-level (n = 84)	Highest-level (n = 101)
	n (%)	n (%)	n (%)	n (%)	n (%)
**Self-rated health**					
Healthy	1818 (76.4%)	319 (69.0%)	153 (65.4%)	52 (61.9%)	58 (57.4%)
Not healthy	433 (18.2%)	105 (22.7%)	65 (27.8%)	25 (29.8%)	32 (31.7%)
Missing data	130 (5.5%)	38 (8.2%)	16 (6.8%)	7 (8.3%)	11 (10.9%)
**Frequency of going outdoors**					
Twice a week or more	2158 (90.6%)	407 (88.1%)	193 (82.5%)	70 (83.3%)	78 (77.2%)
Once a week or less	148 (6.2%)	40 (8.7%)	37 (15.8%)	13 (15.5%)	17 (16.8%)
Missing data	75 (3.1%)	15 (3.2%)	4 (1.7%)	1 (1.2%)	6 (5.9%)
**Chronic disease**					
None	1003 (42.1%)	179 (38.7%)	82 (35.0%)	33 (39.3%)	40 (39.6%)
One or more	1234 (51.8%)	247 (53.5%)	135 (57.7%)	42 (50.0%)	49 (48.5%)
Missing data	144 (6.0%)	36 (7.8%)	17 (7.3%)	9 (10.7%)	12 (11.9%)
**IADL disability**					
None	2107 (88.5%)	390 (84.4%)	187 (79.9%)	68 (81.0%)	77 (76.2%)
One or more	217 (9.1%)	58 (12.6%)	44 (18.8%)	15 (17.9%)	19 (18.8%)
Missing data	57 (2.4%)	14 (3.0%)	3 (1.3%)	1 (1.2%)	5 (5.0%)
**Perceived financial status**					
Not tight	1829 (76.8%)	336 (72.7%)	174 (74.4%)	63 (75.0%)	59 (58.4%)
Tight	484 (20.3%)	110 (23.8%)	58 (24.8%)	19 (22.6%)	36 (35.6%)
Missing data	68 (2.9%)	16 (3.5%)	2 (0.9%)	2 (2.4%)	6 (5.9%)
**Living arrangement**					
Not living alone	1818 (76.4%)	350 (75.8%)	191 (81.6%)	60 (71.4%)	69 (68.3%)
Living alone	493 (20.7%)	93 (20.1%)	39 (16.7%)	22 (26.2%)	24 (23.8%)
Missing data	70 (2.9%)	19 (4.1%)	4 (1.7%)	2 (2.4%)	8 (7.9%)

*IADL* Instrumental Activities of Daily Living.

**Table 2:** Characteristics of participants in follow-up surveys by disengagement levels (N = 3262).

	Zero-level (n = 2381)	Low-level (n = 462)	Middle-level (n = 234)	High-level (n = 84)	Highest-level (n = 101)
**Sex, n (%)**					
Male	1062 (44.6%)	207 (44.8%)	107 (45.7%)	38 (45.2%)	40 (39.6%)
Female	1319 (55.4%)	255 (55.2%)	127 (54.3%)	46 (54.8%)	61 (60.4%)
Age, years, mean [SD]	74.8 [5.3]	75.7 [5.6]	75.7 [5.6]	78.1 [5.8]	76.3 [5.6]
**Self-rated health, n (%)**					
Healthy	1773 (74.5%)	320 (69.3%)	159 (67.9%)	41 (48.8%)	–
Not healthy	425 (17.8%)	101 (21.9%)	69 (29.5%)	28 (33.3%)	
Missing data	183 (7.7%)	41 (8.9%)	6 (2.6%)	15 (17.9%)	
**Frequency of going outdoor, n (%)**					
Twice a week or more	2125 (89.2%)	386 (83.5%)	193 (82.5%)	55 (65.5%)	–
Once a week or less	163 (6.8%)	52 (11.3%)	37 (15.8%)	14 (16.7%)	
Missing data	93 (3.9%)	24 (5.2%)	4 (1.7%)	15 (17.9%)	
Follow-up, months, mean [SD]	91.8 [13.3]	92.5 [12.1]	91.2 [12.8]	86.0 [16.5]	85.2 [18.6]
**Follow-up status, n (%)**					
Survived	1796 (84.3%)	338 (84.5%)	160 (76.9%)	45 (62.5%)	56 (70.0%)
Died	212 (10.0%)	43 (10.8%)	31 (14.9%)	21 (29.2%)	17 (21.3%)
Censored^a^	122 (5.7%)	19 (4.8%)	17 (8.2%)	6 (8.3%)	7 (8.8%)

*IADL* Instrumental Activities of Daily Living, *SD* standard deviation.

^a^Censored participants included those who moved away and refused to respond to the surveys during the follow-up period.

### Association between the disengagement level and health status

After excluding participants with missing data for the covariates and each dependent variable, 2,625 and 2,712 individuals were evaluated for self-rated health and frequency of going outdoors, respectively. In the crude model, participants in the low (odds ratio [OR] 1.41, 95% confidence interval [95% CI] 1.09–1.83), middle (OR .88, 95% CI 1.36–2.58), and high levels (OR 3.69, 95% CI 2.18–6.26) had significantly higher ORs for “not healthy” than those in the zero-level group (Table 3). In the adjusted model, only the high level was significant (OR 3.03, 95% CI 1.56–5.89).

**Table 3:** Association between disengagement levels and negative health status among older adults.

	Crude OR (95% CI)	Adjusted OR (95% CI)
**Self-rated health (FL): not healthy**		
Zero-level	Reference	Reference
Low-level	1.41* (1.09–**1.83)**	1.19 (0.86–1.64)
Middle-level	1.88*** (1.36–**2.58)**	1.29 (0.86–1.94)
High-level	3.69*** (2.18–**6.26)**	3.03** (1.56–**5.89)**
**Frequency of going outdoor (FL): once a week or less**		
Zero-level	Reference	Reference
Low-level	1.91*** (1.33–**2.74)**	1.77** (1.18–**2.66)**
Middle-level	3.04*** (2.03–**4.55)**	1.84* (1.14–**2.98)**
High-level	4.21*** (2.22–**8.00)**	2.71* (1.26–**5.84)**

Adjusted for sex, age at follow-up, self-rated health, frequency of going outdoors, chronic disease, instrumental activities of daily living disability, perceived financial status, and living arrangement at baseline. *FL* follow-up survey, *OR* odds ratio, *CI* confidence interval. Bold numbers are statistically significant (*p < 0.05, **p < 0.01, ***p < 0.001).

Moreover, in the crude model, participants in the low (OR 1.91, 95% CI 1.33–2.74), middle (OR 3.04, 95% CI 2.03–4.55), and high levels (OR 4.21, 95% CI 2.22–8.00) had significantly higher ORs for going outdoors “once a week or less” than those in the zero level. In the adjusted model, ORs increased as the disengagement level increased as follows: low level (OR 1.77, 95% CI 1.18–2.66), middle level (OR 1.84, 95% CI 1.14–2.98), and high level (OR 2.71, 95% CI 1.26–5.84).

### Association between disengagement level and mortality

After excluding the participants with missing data for the covariates, data from 2872 individuals were analyzed. In the crude model, participants in the middle (hazard ratio [HR] 1.55, 95% CI 1.07–2.26), high (HR 3.43, 95% CI 2.19–5.37), and highest levels (HR 2.45, 95% CI 1.49–4.01) had a significantly higher mortality than those in the zero level (Table 4). In the adjusted model, participants in the high (HR 2.11, 95% CI 1.34–3.33) and highest levels (HR 1.92, 95% CI 1.16–3.18) had significant HRs. After excluding older adults who died during the first 6 months of each FL, participants in the middle (HR 1.49, 95% CI 1.01–2.19), high (HR 3.05, 95% CI 1.88–4.93), and highest levels (HR 1.78, 95% CI 1.00–3.19) had significantly higher mortality than those in the zero level (Supplementary Table S1). In the adjusted model, only those in the high-level group had significant HR (HR 1.92, 95% CI 1.16–3.18).

**Table 4:** Association between disengagement levels and mortality among older adults.

	n	Deaths	Incidence per 1000 person-years	Crude HR (95% CI)	Adjusted HR (95% CI)
Zero-level	2119	210	12.9	Reference	Reference
Low-level	397	43	14.0	1.09 (0.78–1.51)	0.97 (0.70–1.35)
Middle-level	208	31	19.7	1.55* (1.07–**2.26)**	1.22 (0.84–1.80)
High-level	71	21	41.5	3.43*** (2.19–**5.37)**	2.11** (1.34–**3.33)**
Highest-level	78	17	30.1	2.45*** (1.49–**4.01)**	1.92* (1.16–**3.18)**

Adjusted for sex, age at follow-up, self-rated health, frequency of going outdoors, chronic disease, instrumental activities of daily living disability, perceived financial status, and living arrangement at baseline. *HR* hazard ratio, *CI* confidence interval. Bold numbers are statistically significant (*p < 0.05, **p < 0.01, ***p < 0.001).

## Discussion

In this study, we operationally defined disengagement from society as dropping out from a longitudinal study and determined the disengagement level based on responses to step-by-step FLs of the dropouts. Examination of the disengagement level, health status, and mortality revealed that higher disengagement levels increased the risk of lower health status in the FL group. Additionally, compared to those who did not disengage, respondents of the home-visit survey and non-respondents, who were at a higher level of disengagement, had twice the mortality risk. Overall, we showed that gradual disengagement from society is associated with negative health outcomes, suggesting an urgent need for efforts to prevent societal disengagement among older adults.

Although many studies have shown that participants who dropped out from longitudinal studies showed lower health status at BL, their health status after BL remains unclear. Our study revealed that, even after adjusting for health status at BL, participant health status at FL was poor among older adults with higher levels of societal disengagement. The proportion of older adults who reported their own health as unhealthy in FLs was high only in those with high-level disengagement who were respondents of the home-visit survey. In contrast, the proportion of homebound individuals who went outdoors once a week or less was significantly higher at all disengagement levels: low, middle, and high. These results suggest that the social aspects of health may have started to decline from a stage with a low disengagement level.

Previous studies have reported that 7–10% of older adults are homebound^20–22^. In this study, 6.8% in the zero-level, 11.3% in the low-level, 15.8% in the middle-level, and 16.7% in the high-level disengagement groups were categorized as homebound at FLs; the higher the level of disengagement, the higher the rates of homebound status identified, than those in previous studies. Homebound older adults were more likely to have depressive symptoms and IADL disability^22^ and were at a high risk of needing long-term care^21^. Moreover, a lower frequency of going out leads to lower functional capacity and intellectual activity^23^. Social participation is essential for achieving successful aging^3–5^. Our findings suggest that promoting participation and preventing homebound status may prevent disengagement from society.

Compared to people who did not disengage, those who were at the high and highest levels had a two-fold higher mortality risk during the 8-year follow-up period. We showed that those with a high level of disengagement had lower self-rated health and a lower frequency of going outdoors, which may be associated with high mortality^24^. This suggests that individuals with the highest level of disengagement should be followed up, as they have an increased risk of mortality. The high mortality risk among dropouts from a longitudinal study who did not respond to home-visit surveys should be considered when conducting a longitudinal study with mortality as an outcome. In our study, no linear proportional relationship was observed between disengagement level and mortality, as the HR of the highest level was slightly lower than that of the high level. In addition, in the sensitivity analysis, only high levels had a significant association with mortality. Participants up to the high level have characteristics of gradually worsening health, while those in the highest-level group may have different characteristics. This result may have been affected by the reasons for non-response. In addition to poor health status, being busy or not interested may be reasons for non-response. A previous study categorized dropouts based on the reason for attrition (impaired, not interested, avoided, or other reasons)^7^; thus, the real situation of disengagement should be clarified by exploring the reasons for non-response.

Our study clarified that, as disengagement from society gradually progresses, health status also gradually declines. In previous studies of older adults, participation was treated as a dichotomous variable (participation or not participation)^3–5^, and no attention was paid to the disengagement level, such as no longer participating or less frequent participation. Future studies focusing on these points should be conducted to further strengthen the knowledge of successful aging. Furthermore, our findings suggest that non-response of older adults who initially responded to the survey may be a useful indicator of poor health status. In the public health field, for example, older adults who cease the use of health and welfare services or health checkups and ignore a guide that encourages their use are considered to be at high risk of negative health outcomes. Thus, careful follow-up, such as home visits, should be performed.

This study had some limitations. First, although we hypothesized that withdrawal from the survey would reflect the nature of disengagement from society and designed and conducted this study to examine this issue, the theoretical justification for this measurement was not confirmed. Therefore, more studies should be conducted to confirm this issue in the future. Second, owing to self-reported health conditions, it was impossible to objectively evaluate the diseases and physical conditions related to negative health status and mortality. Third, our study covered only one urban area of Japan; therefore, our findings cannot be easily generalized to other areas. Fourth, participants’ health attitudes, health literacy, and pro-social attitudes may be confounded but could not be adjusted as these variables were not measured in this study. Fifth, there was a possibility of selection bias because of a complete case analysis. Sixth, although proxy responses by family members were allowed in the home-visit survey, proxy responses were not allowed for self-rated health and frequency of going outdoors, thus, resulting in a large number of missing responses. Nevertheless, this is the first study to investigate the health status of dropouts of a longitudinal study by using step-by-step FLs.

In conclusion, gradual disengagement from society was associated with negative health outcomes, such as poor self-rated health, low frequency of going outdoors, and death. From the perspective of gerontology, maintaining participation in society can lead to good health, reduce mortality, and achieve successful aging. From the perspective of public health, older adults who drop out of surveys and ignore FLs may be at a high risk of poor health. Thus, there is an urgent need to prevent social disengagement among older adults.

## Methods

### Participants

We conducted a mail survey of 7015 residents aged 65–85 years living in nine areas in Itabashi-ku, an urban suburb of Tokyo, Japan. Older adults who were institutionalized residents or had participated in previous surveys conducted by our institute were excluded. The study period was from August to October 2012 (BL), and 3696 residents responded. A follow-up mail survey was administered to these respondents from August to October 2014, and 2381 people answered. For non-respondents, a step-by-step FL, which was simplified and easier to complete, was conducted in sequence. First, for those who did not respond to the mail survey, a simplified mail survey with a reduced number of questionnaire items (from 24 to 10) was conducted from September to October 2015, and 462 people responded. Next, for those who did not respond to the simplified mail survey, a postcard survey with an even smaller number of questionnaire items (five items) was conducted from February to April 2016, and 234 people responded. Finally, for those who did not respond to the postcard survey, a home-visit survey was conducted by examiners visiting the residents in June 2016, and 84 people responded. In total, 434 people moved, refused to respond, or died without a response from the start of the follow-up mail survey to the end of the home-visit survey, and were excluded.

**Figure 1:**
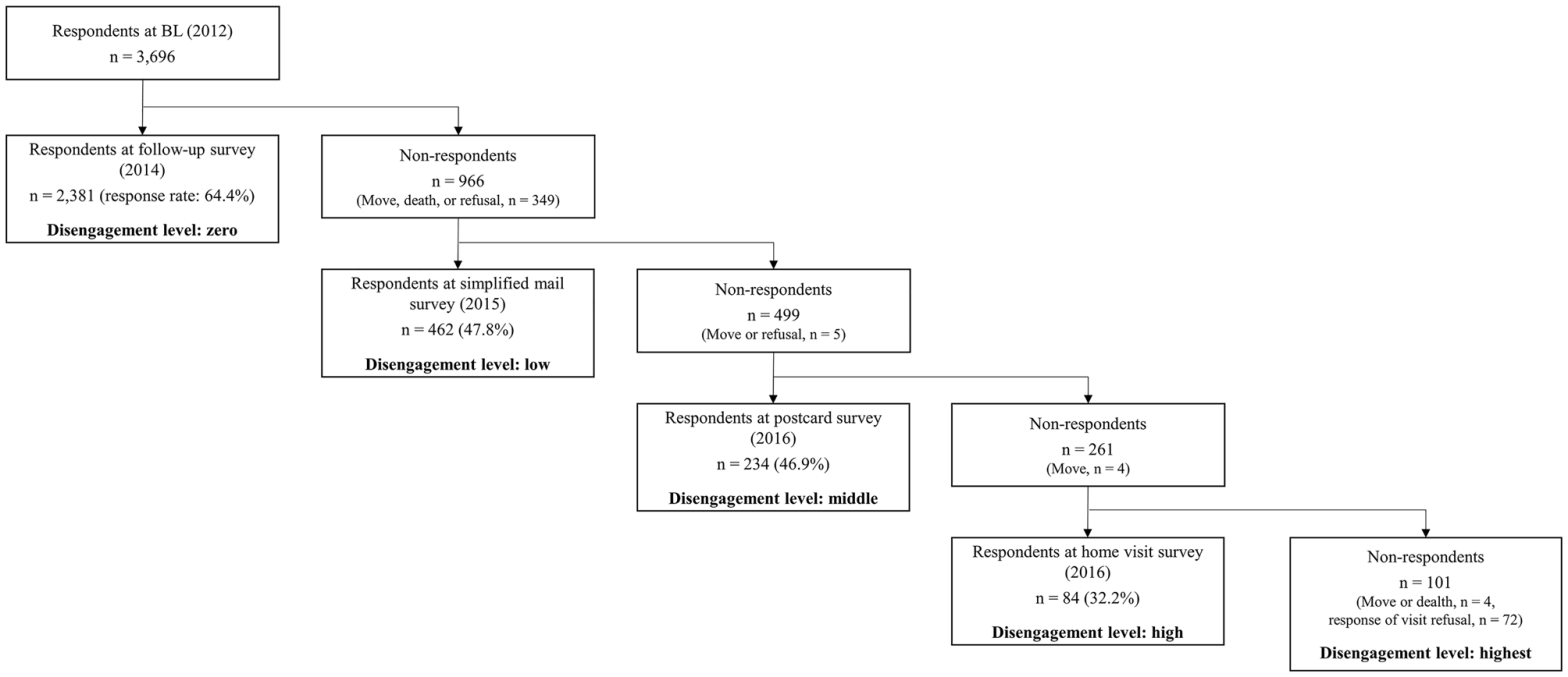
Flow diagram of inclusion and exclusion of participants and stratification by societal disengagement levels.

Disengagement levels were defined as follows, depending on the response to the FLs: zero (mail survey), low (simplified mail survey), middle (postcard survey), high (home-visit survey), and highest (non-respondents to the home-visit survey) (Fig. 1).

Ethical approval was granted by the Ethics Committee of the Tokyo Metropolitan Institute of Gerontology (approval number: 61, 2013). All research was performed in accordance with the Declaration of Helsinki. All participants provided written informed consent to participate in this study.

### Measurements

#### Health status

Self-rated health and frequency of going outdoors were assessed as health status at both the BL and each FL. Each answer at BL was considered a covariate, and FL was the dependent variable. Each of these were converted to dichotomous variables.

With regard to self-rated health, participants were asked, “In general, would you say that your health is good?” The answers were very healthy, sufficiently healthy, not very healthy, and not healthy. Those who answered with very healthy and sufficiently healthy were categorized as healthy, whereas those who reported being not very healthy and not healthy were categorized as not healthy.

With regard to the frequency of going outdoors, participants selected one of the following options: twice a day or more, once a day, once every 2–3 days, once a week, once a month or more, several times a year, and rarely. Based on the definition of homebound, the answers were divided into two categories: twice a week or more and once a week or less^20^.

#### Mortality

Mortality information was obtained from a database administered by the ward office. The follow-up period was from October 1, 2012, to November 1, 2020. This mortality information was based on the notification of death forms for residents.

#### Covariates

The covariates collected at BL were chronic diseases, IADL disability, perceived financial status, and living arrangements. Currently treated chronic diseases were classified into five types: hypertension, stroke, heart disease, diabetes, and cancer. Respondents were categorized as having either none or one or more chronic diseases. IADL was assessed using a subscale of the Tokyo Metropolitan Institute of Gerontology Index of Competence, which includes five questions on instrumental self-maintenance. Respondents were categorized as completely independent or having at least one disability. Perceived financial status was assessed using the following options: very comfortable, comfortable, average, tight, and very tight. Very comfortable to average levels were categorized as not tight, whereas tight and very tight were categorized as tight. Living arrangement was categorized as either living alone or not.

### Statistical analysis

Data on participant characteristics are presented as means and standard deviations for continuous variables and as numbers and percentages for categorical variables.

The relationship between societal disengagement level and health status at FLs was examined by logistic regression analysis with the disengagement level (reference: zero) as the independent variable and self-rated health (not healthy) or frequency of going outdoors (once a week or less) as the dependent variable. We fitted the crude and adjusted models, which were adjusted for sex, age at FLs, self-rated health, frequency of going outdoors, chronic disease, IADL disability, and perceived financial status at BL.

The relationship between disengagement level and mortality was examined using a Cox regression model with disengagement level (reference: zero) as the independent variable. We fitted the crude and adjusted models, which were adjusted for sex, age at FLs, self-rated health, frequency of going outdoors, chronic disease, IADL disability, perceived financial status, and living arrangements at BL. Data regarding the age of participants at FLs with the highest disengagement level were at the end of the home-visit survey. To assess the possibility of reverse causality, older adults who died during the first 6 months of each FL were excluded from sensitivity analyses.

All statistical analyses were performed using IBM SPSS Statistics for Windows version 27 (IBM Japan, Ltd., Tokyo, Japan). Statistical significance was set at *p* < 0.05.

## Supplementary Information

Supplementary Table S1.

## Data Availability

The datasets analyzed during the current study are not publicly available due to ethical and legal restrictions imposed by the Ethics Committee at Tokyo Metropolitan Institute of Gerontology, but are available from the corresponding author on reasonable request.
