# Supplementary material for: Association of social disengagement with health status and all-cause mortality among community-dwelling older adults: evidence from the Otassha study
Source: Sci Rep. 2022 Oct 26;12:17918. doi: 10.1038/s41598-022-22609-y (PMC9606023; doi:10.1038/s41598-022-22609-y)
Supplement: Supplementary file 1 — Supplementary Table S1. [file 41598_2022_22609_MOESM1_ESM.docx]

**Supplementary Table S1: Association between disengagement levels and mortality after excluding older adults who died during the first 6 months of each follow-up**

|  | n | Deaths | Incidence per 1000 person-years | Crude HR (95% CI) | Adjusted HR (95% CI) |
| --- | --- | --- | --- | --- | --- |
| Zero-level | 2,115 | 206 | 12.6 | Reference | Reference |
| Low-level | 393 | 39 | 12.8 | 1.01 (0.71–1.42) | 0.90 (0.64–1.27) |
| Middle-level | 205 | 29 | 18.6 | **1.49* (1.01**–**2.19)** | 1.16 (0.78–1.73) |
| High-level | 68 | 18 | 36.5 | **3.05*** (1.88**–**4.93)** | **1.88** (1.15**–**3.06)** |
| Highest-level | 73 | 12 | 21.9 | **1.78* (1.00**–**3.19)** | 1.40 (0.78–2.53) |

*Note.* Adjusted for sex, age at follow-up, self-rated health, frequency of going outdoors, chronic disease, instrumental activities of daily living disability, perceived financial status, and living arrangement at baseline. HR: hazard ratio; CI: confidence interval. Bold numbers are statistically significant (**p* <0.05, ***p* <0.01, ****p* <0.001).
